# Are IDH1 R132 Mutations Associated With Poor Prognosis in Patients With Chondrosarcoma of the Bone?

**DOI:** 10.1097/CORR.0000000000002960

**Published:** 2024-01-03

**Authors:** Giulia Trovarelli, Marta Sbaraglia, Andrea Angelini, Elena Bellan, Elisa Pala, Elisa Belluzzi, Assunta Pozzuoli, Chiara Borga, Angelo Paolo Dei Tos, Pietro Ruggieri

**Affiliations:** 1 Department of Orthopedics and Orthopedic Oncology, University of Padua, Padua, Italy; 2 Department of Surgery, Oncology and Gastroenterology of University of Padova, Padua, Italy; 3 Surgical Pathology and Cytopathology Unit, Department of Medicine-DIMED, University of Padua School of Medicine, Padua, Italy

## Abstract

**Background:**

Because chondrosarcomas vary widely in their behavior, and because anticipating their behavior based on histology alone can be challenging, genetic markers represent an appealing area of inquiry that may help us refine our prognostic approaches. Isocitrate dehydrogenase (IDH) mutations are involved in the pathogenesis of a variety of neoplasms, and recently, IDH1/2 mutations have been found in the tissue of benign cartilage tumors as well as in conventional chondrosarcomas and highly aggressive dedifferentiated chondrosarcomas. However, their association with patient survival is still controversial.

**Questions/purposes:**

(1) What proportion of patients with chondrosarcomas carry IDH mutations, and which IDH mutations can be found? (2) Are any specific IDH mutations associated with poorer overall survival, metastasis-free survival, or local recurrence-free survival?

**Methods:**

Between April 2017 and December 2022, we treated 74 patients for atypical cartilaginous tumors or chondrosarcomas in a musculoskeletal tumor referral center. Patients were considered potentially eligible for the present study if the histologic diagnosis was confirmed by two expert soft tissue and bone pathologists following the current WHO classification, complete preoperative imaging and follow-up data were available, surgical excision was performed by sarcoma orthopaedic surgeons directed by a team leader, and the minimum follow-up was 2 years after surgical treatment unless the patient died. Data including sex, age, diagnosis, grade, type of operation, local recurrence, metastasis, and oncologic follow-up were recorded. Forty-one patients (55%) were eligible for the study. For each patient, DNA was extracted and quantified from paraffin-embedded sections of tumor tissue, and the mutational status of IDH1 (codons 105 and 132) and IDH2 (codons 140 and 172) genes was assessed. Of those, 56% (23 of 41) of patients had adequate DNA for analysis of IDH mutations: 10 male and 13 female patients, with a median age of 59 years (range 15 to 98 years). There were 22 conventional chondrosarcomas (8 atypical cartilaginous tumors, 11 Grade 2, and 3 Grade 3) and 1 dedifferentiated chondrosarcoma. Stage was IA in 3 patients, IB in 5, IIA in 1, IIB in 13, and III in 1, according to the Musculoskeletal Tumor Society classification. At a median follow-up of 3.5 years (range 4 months to 5.6 years), 14 patients were disease-free, 2 were alive with disease, and 7 died (3 within 2 years from surgery). Eight patients had metastases, and 7 developed local recurrence. We determined the proportion of patients who carried IDH mutations, and compared patients with and without those mutations in terms of overall survival, metastasis-free survival, and local recurrence-free survival using Kaplan-Meier curves.

**Results:**

Six patients showed wild-type IDH genes, and 17 had IDH mutations (12 had IDH1 R132, 3 had IDH1 G105, and 2 had IDH2 R172). Overall survival at 2 years using the Kaplan-Meier estimator was lower in patients with an IDH mutation than in those with the wild-type gene (75% [95% confidence interval 50% to 99%] versus 100% [95% CI 100% to 100%]; p = 0.002). Two-year metastasis-free survival was also lower in patients with an IDH mutation than in those with the wild-type gene (33% [95% CI 7% to 60%] versus 100% [95% CI 100% to 100%]; p = 0.001), as was 2-year local recurrence-free survival (70% [95% CI 42% to 98%] versus 100% [95% CI 100% to 100%]; p = 0.02).

**Conclusion:**

We found that IDH1 R132 mutations were negatively associated with the prognosis of patients with bone chondrosarcomas. Nevertheless, more extensive studies (such as multicenter international studies) are needed and advisable to confirm our observations in this preliminary small series. Moreover, evaluating mutational status in fresh samples instead of in paraffin-embedded sections could help to increase the number of patients with adequate DNA for analysis. If our findings will be confirmed, the evaluation of IDH mutational status in biopsy samples or resection specimens could be considered when stratifying patients, highlighting those who may benefit from more aggressive treatment (such as adjuvant chemotherapy) or closer follow-up.

**Level of Evidence:**

Level III, prognostic study.

## Introduction

Chondrosarcoma is a primary malignant bone tumor characterized by abundant cartilage matrix production; its clinical behavior depends mainly on the histologic grade, with higher-grade tumors associated with poorer survival [4, 6, 39, 41]. Chondrosarcoma is generally thought to be resistant to chemotherapy and radiotherapy because of its slow growth rate with a relatively low fraction of dividing cells; consequently, surgery is believed to be the only available curative option [5, 12, 17, 33]. However, despite appropriate surgical treatment, there is a high risk of local recurrence and metastases in Grades 2 and 3 chondrosarcomas, and these events decrease overall survival [3, 4, 6, 39, 41]. Thus, predicting the clinical outcomes and which patients are at increased risk of relapse based only on histologic grade can be challenging [3, 4, 19, 24]. Therefore, current research has focused on identifying new therapeutic targets and potential predictive markers, including genetic ones, that may help orthopaedic surgeons refine prognostic approaches [7, 10, 15, 29, 34, 36-38].

Specific driver mutations in isocitrate dehydrogenase (IDH) have been identified in a variety of neoplasms, including cholangiocarcinoma; acute myeloid leukemia; gliomas; thyroid, breast, and prostate carcinomas; and melanoma [14, 18, 25, 27, 31, 37, 42, 43, 46, 47, 49]. Their potential role as prognostic and predictive markers has been investigated: IDH1/2 mutations do not implicate alternative treatment or additional prognostically impactful information in most of the above diseases, whereas IDH1/2 mutations may lead to treatment with biologic actives in IDH1/2-mutated intrahepatic cholangiocarcinoma or differential diagnosis in neuropathology [14, 18, 25, 27, 31, 37, 42, 43, 46, 47, 49]. IDH is a β-decarboxylating dehydrogenase enzyme in the tricarboxylic acid cycle and is implicated in the synthesis of nucleotides, lipids, and amino acids [21]. Three isoforms have been identified: IDH1 in the cytoplasm and peroxisomes and IDH2 and IDH3 in mitochondria [35]. Mutated IDH enzymes produce D-2-hydroxyglutarate oncometabolite [11], which accumulates in cells, leading to histone and DNA hypermethylation and impairing normal cell differentiation and promoting tumorigenesis [8, 26]. IDH mutations also influence other cellular processes, including metabolism, cell growth signaling pathways, and DNA damage repair [30, 41, 45]. These genetic mutations are missense substitutions that most commonly occur in codons R132 or G105 of IDH1 and codons R172 or R140 of the IDH2 gene [11, 20, 48].

Recently, IDH1/2 mutations were found in the tumor tissue of benign cartilage tumors and in conventional chondrosarcoma and highly aggressive dedifferentiated chondrosarcomas [1, 2, 7, 9, 22, 23, 28, 34, 39, 40, 50]; however, their association with prognosis remains controversial [1, 9, 28, 32, 51], such as their use in patient treatment [10].

We therefore asked: (1) What proportion of patients with chondrosarcomas carry IDH mutations, and which IDH mutations can be found? (2) Are any specific IDH mutations associated with poorer overall survival, metastasis-free survival, or local recurrence-free survival?

## Patients and Methods

### Study Design and Setting

This was a prospective study in which IDH mutational status was analyzed in patients who underwent surgery for atypical cartilaginous tumors (ACTs) or chondrosarcoma in an urban musculoskeletal tumor referral center.

The inclusion criteria were confirmed histologic diagnosis of chondrosarcoma by two expert soft tissue and bone pathologists (APDT and MS) in accordance with the current WHO classification [44], complete preoperative imaging and follow-up data, surgical excision of the chondrosarcoma by sarcoma orthopaedic surgeons (AA, EP, and GT) directed by a team leader (PR), and minimum follow-up of 2 years after surgical treatment unless the patient died. Data regarding sex, age, histologic diagnosis, site, pathologic fracture, type of surgery, margins, local recurrence, and metastasis were examined.

### Participants

**Fig. 1:**
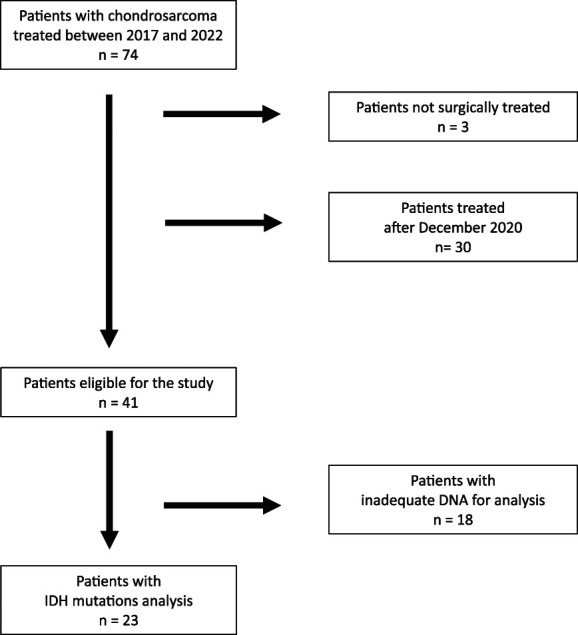
This flow diagram describes our inclusion and exclusion criteria for patients enrolled in the study.

Between April 2017 and December 2022, we treated 74 patients. Thirty-three patients were excluded from the analysis because they did not receive surgical treatment (3 patients) or because they were treated after 2020 (30 patients), with a minimum follow-up shorter than 2 years (Fig. 1).

All patients had staging and fluoroscopy-guided trocar biopsy (8-gauge) before surgery. The surgical treatment was as follows: extensive curettage and filling of the bone defect with polymethyl methacrylate for appendicular ACT, considering the expected low risk of metastasis and local recurrence; wide resection for ACT with aggressive radiologic findings (such as cortex expansion with cortical disruption) or massive metaphyseal involvement, as well as Grade 2 or 3 chondrosarcoma and dedifferentiated chondrosarcoma; and amputation for high-grade chondrosarcoma when neurovascular structures were involved, precluding functional limb salvage. After surgery, specimens were evaluated by the same pathologists (APDT and MS) who analyzed the biopsy samples. All analyses in this study were based on the final pathology report.

### Description of Experiment, Treatment, or Surgery

Forty-one patients (55%) were eligible for the analysis of IDH mutational status. For each patient, five 10-µm paraffin-embedded sections of resected tumor were used to extract DNA using the QIAmp FFPE tissue Kit (Qiagen) according to the manufacturer’s instructions. Extracted DNA was quantified using the Qubit® 3.0 fluorometer and the Qubit® DNA BR Assay kit (Thermo Fisher Scientific). The mutational status of IDH1 (codons 105 and 132) and IDH2 (codons 140 and 172) was assessed using the kit EasyPGX ready IDH1-2, according to the manufacturer’s instructions (Diatech Pharmacogenetics). This real-time polymerase chain reaction–based assay allows the coamplification of mutated alleles (FAM-labeled probes) and an endogenous control gene (HEX-labeled probes). Amplifying the control gene allows for an evaluation of DNA quality. For each reaction, 15 to 30 ng of DNA were used. Results were analyzed using the EasyPGX analysis software.

### Descriptive Data

A total of 56% of patients (23 of 41) had adequate tissue and DNA for the analysis of IDH mutations (Table 1), including 10 male and 13 female patients with a median age of 59 years (range 15 to 98 years). There were 22 conventional chondrosarcoma (8 ACT, 11 Grade 2, 3 Grade 3), and 1 dedifferentiated chondrosarcoma. The most frequent site was the lower limb (11 patients), followed by the upper limb (eight) and the periacetabular area (four). No patient had a pathologic fracture, and one presented with lung metastases at the time of diagnosis. The stage was IA in 3 patients, IB in five, IIA in one, IIB in 13, and III in one, according to the Muscoluskeletal Tumor Society classification. Surgery at our department was the first treatment for all patients except for four patients, who were treated for local recurrence of a previously managed chondrosarcoma with inadequate margins at another center. Surgical margins were adequate in all patients: wide for all resections (16) and amputations (4) and intralesional by choice in patients with ACTs treated with curettage (3). None of the patients received adjuvant treatments, except one with multiple lung metastases who started palliative chemotherapy with cisplatin, doxorubicin, and ifosfamide according to the EUROpean Bone Over 40 Sarcoma Study protocol [16].

**Table 1.** Demographic and oncologic data of patients with chondrosarcoma in whom the IDH mutational status was analyzed (n = 23)

Patient	Gender	Age in years	Grade	Site	Stage	Type of surgery	IDH mutations	Metastases	LR	Outcome	Follow-up in months
1	W	98	Grade 2	Foot	IIB	Amputation	IDH1 R132	No	No	NED	40
2	M	15	Grade 2^a^	Pelvis	IIB	Resection	Wild-type	No	Yes	NEDrl	68
3	M	64	Grade 2	Scapula	IIB	Resection	IDH1 R132	No	No	NED	66
4	W	59	ACT	Humerus	IB	Resection	IDH1 G105	No	No	NED	65
5	W	61	ACT^a^	Calcaneus	IB	Resection	Wild-type	No	No	NED	65
6	M	76	Grade 3	Femur	IIB	Resection	IDH2 R172	No	No	NED	62
7	M	45	Grade 2^a^	Femur	IIB	Resection	Wild-type	No	Yes	NEDrl	62
8	M	69	Grade 2	Pelvis	IIB	Amputation	IDH1 R132	Yes	Yes	DWD	17
9	W	56	Grade 2	Femur	III	Amputation	IDH1 R132	Yes	No	DWD	31
10	W	69	Grade 2^a^	Tibia	IIB	Resection	IDH1 R132	Yes	No	DWD	4
11	M	38	Grade 2	Pelvis	IIB	Resection	IDH1 R132	Yes	Yes	DWD	39
12	W	67	ACT	Femur	IB	Resection	Wild-type	No	No	NED	53
13	W	36	Grade 2	Femur	IIB	Resection	IDH1 R132	Yes	Yes	AWD	48
14	W	55	Grade 3	Fibula	IIB	Resection	IDH1 R132	Yes	Yes	AWD	47
15	M	21	ACT	Femur	IB	Resection	Wild-type	No	No	NED	46
16	W	89	Dedifferentiated	Humerus	IIB	Amputation	IDH1 R132	Yes	No	DWD	4
17	W	82	Grade 3	Scapula	IIB	Amputation	IDH1 R132	Yes	No	DWD	43
18	W	61	ACT	Humerus	IB	Resection	IDH1 G105	No	No	NED	39
19	M	58	ACT	Acromion	IA	Curettage	Wild-type	No	No	NED	39
20	W	44	ACT	Humerus	IA	Curettage	IDH1 R132	No	No	NED	38
21	M	43	Grade 2	Femur	IIA	Resection	IDH1 G105	No	No	NED	37
22	W	20	ACT	Humerus	IA	Curettage	IDH2 R172	No	No	NED	34
23	M	65	Grade 2	Pelvis	IIB	Resection	IDH1 R132	No	Yes	DWD	29

a Patients who had previous inadequate surgical treatment elsewhere. IDH = isocitrate dehydrogenase; LR = local recurrence; M = man; W = woman; ACT = atypical cartilaginous tumors; NED = no evidence of disease; AWD = alive with disease; DWD = dead with disease; NEDrl = no evidence of disease after treatment of local recurrence.

### Description of Follow-up Routine

The follow-up protocol included physical examinations, radiographs, and MRI with contrast of the treated site every 3 months during the first 3 years, every 4 months in the fourth year, every 6 months in the fifth year, and then annually for the other 5 years. Lung CT was performed at every examination of patients with high-grade chondrosarcomas and every 6 months in those with ACT. No patient was lost to follow-up. At a median follow-up of 3.5 years (range 4 months to 5.6 years), seven patients had local recurrence and eight had lung metastases. Local recurrence occurred at a median of 23 months (range 12 to 30) after surgery and was treated with wide excision (three patients) or amputation (two), while two patients were considered inoperable. Seven patients developed new lung metastases at a median of 12 months after surgery (range 2 to 22); three of them received palliative chemotherapy according to the EUROpean Bone Over 40 Sarcoma Study protocol. The others received no therapy owing to their poor general condition; these patients died after a few months. Four patients had local recurrences and metastases. At the last follow-up examination, 14 patients were disease-free, two were alive with disease, and seven died (three within 2 years from surgery).

### Primary and Secondary Study Outcomes

Our primary study goal was to determine the proportion of patients who carried IDH mutations. Our secondary study goal was to compare the oncologic outcomes of patients with and without those mutations, analyzing local recurrence, metastasis, or death.

### Statistical Analysis

We analyzed overall survival as the time from surgery to the last follow-up or death, local recurrence-free survival as the time from surgery to local recurrence, and metastases-free survival as the time from surgery to metastasis. Considering that positive surgical margins are a possible prognostic factor that influences the recurrence rate, we decided to exclude the subgroup of patients previously treated elsewhere with inadequate margins from the analysis. Survival curves were analyzed using a Kaplan-Meier analysis, and the curves were compared using a log-rank test; p values less than 0.05 were considered significant. Because of the limited number of patients in our series, we did not perform a multivariate analysis.

## Results

### Proportion of Patients With IDH Mutations and Mutation Types

Of the 23 patients, six had wild-type IDH1/2 genes and 17 had IDH1/2 mutations: 12 were IDH1 R132, three were IDH1 G105, and two were IDH2 R172. IDH1 R132 mutations occurred in all grades (Table 2). Four patients with Grade 1 chondrosarcomas had IDH1/2 mutations, whereas nine patients with Grade 2 chondrosarcomas had IDH1 mutations. All patients with Grade 3 chondrosarcomas and dedifferentiated chondrosarcomas had IDH1/2 mutations.

**Table 2.** IDH mutations found in 23 patients with chondrosarcoma and their correlation with oncologic outcomes

	Wild-type	IDH1 G105	IDH2 R172	IDH1 R132
Diagnosis				
Grade 1 chondrosarcoma	4	2	1	1
Grade 2 chondrosarcoma	2	1		8
Grade 3 chondrosarcoma			1	2
Dedifferentiated chondrosarcoma				1
Stage				
IA	1		1	1
IB	3	2		
IIA		1		
IIB	2		1	10
III				1
Oncologic outcomes				
No evidence of disease	6	3	2	3
Alive with disease				2
Dead with disease				7
Relapse of disease				
Local recurrence	2			5
Metastases				8 (1 at the time of diagnosis)

IDH = isocitrate dehydrogenase.

### Are IDH Mutations Associated With Poorer Survival?

Although the numbers of patients with each mutation were small and generally precluded a statistical analysis, patients with IDH R132 did especially poorly (Table 2).

**Fig. 2:**
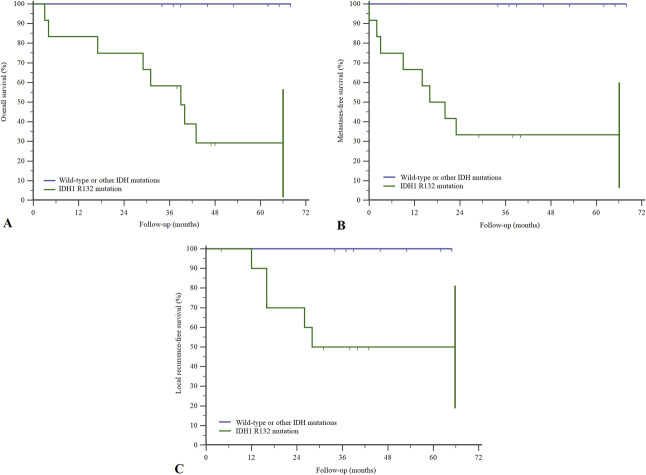
Kaplan-Meier survival curves show that (**A**) overall survival was lower in patients with the IDH1 R132 mutation (p = 0.002). Similarly, (**B**) metastases-free survival (p = 0.001) and (**C**) local recurrence-free survival (p = 0.02) were also lower.

**Fig. 3:**
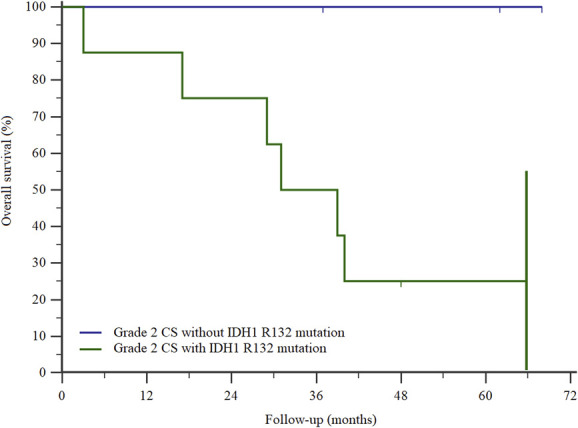
This Kaplan-Meier survival curve shows lower overall survival in patients affected by Grade 2 chondrosarcoma if they also carried the IDH1 R132 mutation (p = 0.04).

Overall survival at 2 years using the Kaplan-Meier estimator was lower in patients with an IDH mutation than in those with the wild-type gene (75% [95% confidence interval 50% to 99%] versus 100% [95% CI 100% to 100%]; p = 0.002) (Fig. 2A). Two-year metastasis-free survival was also lower (33% [95% CI 7% to 60%] versus 100% [95% CI 100% to 100%]; p = 0.001) (Fig. 2B), as was 2-year local recurrence-free survival (70% [95% CI 42% to 98%] versus 100% [95% CI 100% to 100%]; p = 0.02) (Fig. 2C). Patients with Grade 2 chondrosarcoma had lower 2-year overall survival if they also presented with the IDH1 R132 mutation than those with the wild-type gene or other mutations (75% [95% CI 45% to 100%] versus 100% [95% CI 100% to 100%]; p = 0.04) (Fig. 3). With the numbers we had, we could not show differences among patients with other chondrosarcoma grades.

## Discussion

Chondrosarcomas show wide morphologic and clinical heterogeneity; consequently, the prediction of their behavior cannot be based only on histologic features [3, 4, 19, 24]. Therefore, identifying new prognostic markers is advisable, and among these, genetic markers could be a useful tool that may help surgeons refine their prognostic approaches [7, 10, 15, 29, 34, 36-38]. IDH mutations are involved in the pathogenesis and prognosis of a variety of neoplasms, and recently, IDH1/2 mutations have been found in cartilage tumors [1, 2, 7, 9, 22, 23, 28, 34, 39, 40, 50]. These genomic alterations can have a meaningful diagnostic impact and be a potential therapeutic target. IDH mutations help discriminate between dedifferentiated chondrosarcoma and other bone sarcomas [7, 13], and trials with IDH inhibitors are ongoing [10]. Because the association between IDH mutations and the survival of patients with chondrosarcomas is still controversial [1, 9, 22, 28, 32, 50, 51], we decided to explore the pattern of IDH mutations in patients with chondrosarcoma and its relationship with clinical outcomes. Our preliminary results demonstrated a poor prognosis in patients carrying IDH1 R132 mutations. If the results are confirmed in large series, the management of chondrosarcomas might be improved.

### Limitations

The main limitation of our series is the small number of patients evaluated. Indeed, the small sample size precludes performing a statistical analysis of the different subgroups, preventimg us from assessing possible differences in age and gender or detecting relationships between other mutations and overall survival, metastasis-free survival, and recurrence-free survival. Moreover, we had insufficient numbers for a multivariable analysis, which could be helpful to remove confounding variables such as tumor grade. However, in this preliminary study, we found that all different mutations can occur in all grades, and patients harboring IDH1 R132 mutations showed lower overall survival, metastasis-free survival, and recurrence-free survival than those with wild-type or alternative mutations. The grade was a prognostic factor; thus, we also analyzed the influence of IDH mutational status according to grade. We found that patients with Grade 2 had worse prognoses when carrying IDH1 R132 mutations. Therefore, the IDH1 R132 mutation seems to be an additional prognostic factor beyond the grade.

Second, the follow-up period was relatively short, and some patients may have recurrences or cancer-related deaths. However, local recurrence and metastases most frequently occur within 2 years after surgery in patients with high-grade chondrosarcoma, and all our patients had a minimum follow-up of 2 years (except those who died before the 2-year interval owing to disease progression). Thus, although the follow-up is short, we believe it to be adequate for the purpose of the study. Nevertheless, additional follow-up studies are essential to validate the results reported herein.

Third, we did not include patients with enchondromas, in which IDH1 and IDH2 mutations can also be detected. However, we think this is not a disqualifying aspect of our analysis because we included patients with ACTs; in the extremities, ACTs have biological behavior more similar to that of enchondromas than to high-grade chondrosarcoma.

Finally, this study provides information about the mutational status of a smaller subset of patients than the initially selected cohort. Indeed, in 18 of 44 eligible patients, the tissue quality for IDH1/2 mutational analysis was affected by decalcification products before paraffin embedding and variability in cellularity and components of the cartilaginous matrix. Although 18 patients were excluded from the molecular evaluation, the results that support the association between the IDH1 R132 mutation and poor prognosis are important. Even the implementation of molecular IDH1/2 profiling in routine clinical practice will remain challenging. Evaluating mutational status in fresh samples instead of paraffin-embedded sections could help to increase the number of patients with adequate DNA for analysis.

### Proportion of Patients With IDH Mutations and Mutation Types

In our series, IDH mutations were present in 17 of 23 patients regardless of grade, and the IDH1 R132 mutation was the most frequently encountered. IDH1/2 mutations accounted for 13 of 15 high-grade chondrosarcomas. Other retrospective studies reported IDH mutations (mainly IDH1 R132 followed by IDH2 R172) in 34% to 67% of patients with chondrosarcomas, more frequently in high-grade than in low-grade chondrosarcomas [1, 9, 28, 32, 51] (Table 3).

**Table 3.** Other studies about IDH mutations in chondrosarcoma

Study	Setting	Number of patients (range of time)	IDH mutations	Association with survival
Amary et al. [1]	Multicentric Retrospective	138 (not reported)	54%	No association with survival
			IDH1 R132	
			IDH2 R172	
Cleven et al. [9]	Retrospective	67 (not reported)	67%	No association with survival
Lugowska et al. [28]	Retrospective	80 (1996-2013)	34%	Overall survival lower in IDH mutations
			IDH1 R132	
			IDH2 R172	
			IDH2 R140	
Zhu et al. [51]	Retrospective	79 (1984-2017)	45%	Time to metastasis longer in IDH mutations, but no difference in MFS
			IDH1 R132	
			IDH2 R172	
Nakagawa et al. [32]	Retrospective	38 (1995-2015)	52%	IDH mutations were associated with poor survival
			IDH1 R132	
			IDH2 R172	
Current study	Prospective	23 (2017-2020)	74%	IDH1 R132 mutations associated with worse OS, MFS, and RFS
			IDH1 R132	
			IDH1 G105	
			IDH2 R172	

IDH = isocitrate dehydrogenase; MFS = metastases-free survival; OS = overall survival; RFS = local recurrence-free survival.

Although we could define the IDH mutational status in only approximately half of the patients (56% [23 of 41]), the proportion of patients with IDH mutations is similar to those already reported. Therefore, we think the results may be applied to the general population of patients with chondrosarcomas.

### Are IDH Mutations Associated With Poorer Survival?

We found poorer overall survival, metastasis-free survival, and recurrence-free survival in patients with the IDH1 R132 mutation. Other more extensive series documented discordant results [1, 9, 28, 32, 51]. Some authors [28, 32] reported poor prognoses in patients with IDH mutations, whereas others [1, 9] found no differences in survival in patients with IDH mutations and those without. Lastly, one other study [51] noted that the time to metastasis was longer in patients with IDH mutations than in those without (50 versus 19 months in high-grade chondrosarcoma and 16 versus 5.5 months in dedifferentiated chondrosarcoma), although metastasis-free survival was no different. The conflicting results of others could be related to the fact that patients’ data were retrospectively analyzed and collected over a long period or from different institutions, and sometimes data about the type of surgery performed and surgical margins obtained were lacking. Moreover, other authors considered the presence of the IDH1/2 mutation (considering all mutations) without analyzing the association between the different types and prognosis. Conversely, our study, although limited by the number of patients, was more homogeneous and included prospectively collected data from patients who were treated over a short period by the same surgical team (with the same level of expertise) who obtained oncologically adequate surgical margins in all patients. In addition, we separately examined the different IDH mutations.

### Conclusion

IDH mutational status, specifically IDH1 R132 mutations, is associated with poorer overall survival in patients with chondrosarcoma. These preliminary results must be confirmed in a larger group of patients. Multicenter, international studies could be helpful. Moreover, evaluating the mutational status in fresh samples instead of in paraffin-embedded sections could help to increase the number of patients with adequate DNA for analysis. If our findings are confirmed, the evaluation of IDH mutational status in biopsy samples or resection specimens could be considered when stratifying patients, highlighting those who may benefit from more aggressive treatment than simple curettage, even in patients with low-grades chondrocarcomas. Novel agents may be discovered or chemotherapy might be considered for patients with high-grade chondrosarcomas and an IDH mutation, or a more frequent follow-up schedule might be considered to detect relapses early.

## References

[1] Amary MF Bacsi K Maggiani F IDH1 and IDH2 mutations are frequent events in central chondrosarcoma and central and periosteal chondromas but not in other mesenchymal tumours. J Pathol. 2011;224:334-343. 21598255 10.1002/path.2913

[2] Amary MF Ye H Forbes G Isocitrate dehydrogenase 1 mutations (IDH1) and p16/CDKN2A copy number change in conventional chondrosarcomas. Virchows Arch. 2015;466:217-222. 25432631 10.1007/s00428-014-1685-4 PMC4325180

[3] Andreou D Gilg MM Gosheger G Metastatic potential of grade I chondrosarcoma of bone: results of a multi-institutional study. Ann Surg Oncol. 2016;23:120-125. 26350369 10.1245/s10434-015-4852-1

[4] Angelini A Guerra G Mavrogenis AF Pala E Picci P Ruggieri P. Clinical outcome of central conventional chondrosarcoma. J Surg Oncol. 2012;106:929-937. 22649023 10.1002/jso.23173

[5] Boehme KA Schleicher SB Traub F Rolauffs B. Chondrosarcoma: a rare misfortune in aging human cartilage? The role of stem and progenitor cells in proliferation, malignant degeneration and therapeutic resistance. Int J Mol Sci. 2018;19:311. 29361725 10.3390/ijms19010311 PMC5796255

[6] Bus MPA Campanacci DA Albergo JI Conventional primary central chondrosarcoma of the pelvis: prognostic factors and outcome of surgical treatment in 162 patients. J Bone Joint Surg Am. 2018;100:316-325. 29462035 10.2106/JBJS.17.00105

[7] Chen S Fritchie K Wei S Diagnostic utility of IDH1/2 mutations to distinguish dedifferentiated chondrosarcoma from undifferentiated pleomorphic sarcoma of bone. Hum Pathol. 2017;65:239-246. 28552826 10.1016/j.humpath.2017.05.015

[8] Chowdhury R Yeoh KK Tian YM The oncometabolite 2-hydroxyglutarate inhibits histone lysine demethylases. EMBO Rep. 2011;12:463-469. 21460794 10.1038/embor.2011.43 PMC3090014

[9] Cleven AHG Suijker J Agrogiannis G IDH1 or -2 mutations do not predict outcome and do not cause loss of 5-hydroxymethylcytosine or altered histone modifications in central chondrosarcomas. Clin Sarcoma Res. 2017;7:8. 28484589 10.1186/s13569-017-0074-6 PMC5418698

[10] Cojocaru E Wilding C Engelman B Huang P Jones RL. Is the IDH mutation a good target for chondrosarcoma treatment? Current Molecular Biology Reports. 2020;6:1-9.

[11] Dang L White DW Gross S Cancer-associated IDH1 mutations produce 2-hydroxyglutarate. Nature. 2010;465:966. 20559394 10.1038/nature09132 PMC3766976

[12] David E Blanchard F Heymann MF The bone niche of chondrosarcoma: a sanctuary for drug resistance, tumour growth and also a source of new therapeutic targets. Sarcoma. 2011;2011:932451. 10.1155/2011/932451 PMC3103994 21647363

[13] de Andrea CE San-Julian M Bovée J. Integrating morphology and genetics in the diagnosis of cartilage tumors. Surg Pathol Clin. 2017;10:537-552. 28797501 10.1016/j.path.2017.04.005

[14] Fathi AT Sadrzadeh H Comander AH Isocitrate dehydrogenase 1 (IDH1) mutation in breast adenocarcinoma is associated with elevated levels of serum and urine 2-hydroxyglutarate. Oncologist. 2014;19:602-607. 24760710 10.1634/theoncologist.2013-0417 PMC4041671

[15] Fede C Stecco C Angelini A Variations in contents of hyaluronan in the peritumoral micro-environment of human chondrosarcoma. J Orthop Res. 2019;37:503-509. 30444002 10.1002/jor.24176

[16] Ferrari S Bielack SS Smeland S EURO-B.O.S.S.: a European study on chemotherapy in bone-sarcoma patients aged over 40: outcome in primary high-grade osteosarcoma. Tumori. 2018;104:30-36. 29218692 10.5301/tj.5000696

[17] Gelderblom H Hogendoorn PC Dijkstra SD The clinical approach towards chondrosarcoma. Oncologist. 2008;13:320-329. 18378543 10.1634/theoncologist.2007-0237

[18] Ghiam AF Cairns RA Thoms J IDH mutation status in prostate cancer. Oncogene. 2012;31:3826. 22120718 10.1038/onc.2011.546

[19] Grimer RJ Gosheger G Taminiau A Dedifferentiated chondrosarcoma: prognostic factors and outcome from a European group. Eur J Cancer. 2007;43:2060-2065. 17720491 10.1016/j.ejca.2007.06.016

[20] Gross S Cairns RA Minden MD Cancer-associated metabolite 2-hydroxyglutarate accumulates in acute myelogenous leukemia with isocitrate dehydrogenase 1 and 2 mutations. J Exp Med. 2010;207:339-344. 20142433 10.1084/jem.20092506 PMC2822606

[21] Haselbeck RJ McAlister-Henn L. Function and expression of yeast mitochondrial NAD- and NADP-specific isocitrate dehydrogenases. J Biol Chem. 1993;268:12116-12122. 8099357

[22] Hirata M Sasaki M Cairns RA Mutant IDH is sufficient to initiate enchondromatosis in mice. Proc Natl Acad Sci U S A. 2015;112:2829-2834. 25730874 10.1073/pnas.1424400112 PMC4352794

[23] Jin Y Elalaf H Watanabe M Mutant IDH1 dysregulates the differentiation of mesenchymal stem cells in association with gene-specific histone modifications to cartilage- and bone-related genes. PLoS One. 2015;10:e0131998. 10.1371/journal.pone.0131998 PMC4498635 26161668

[24] Kamal AF Husodo K Prabowo Y Hutagalung EU. Correlation between survival and tumour characteristics in patients with chondrosarcoma. J Orthop Surg (Hong Kong). 2015;23:365-369. 26715720 10.1177/230949901502300323

[25] Kang MR Kim MS Oh JE Mutational analysis of IDH1 codon 132 in glioblastomas and other common cancers. Int J Cancer. 2009;125:353-355. 19378339 10.1002/ijc.24379

[26] Koivunen P Lee S Duncan CG Transformation by the (R)-enantiomer of 2-hydroxyglutarate linked to EGLN activation. Nature. 2012;483:484-488. 22343896 10.1038/nature10898 PMC3656605

[27] Lopez GY Reitman ZJ Solomon D IDH1(R132) mutation identified in one human melanoma metastasis, but not correlated with metastases to the brain. Biochem Biophys Res Commun. 2010;398:585-587. 20603105 10.1016/j.bbrc.2010.06.125 PMC2987603

[28] Lugowska I Teterycz P Mikula M IDH1/2 mutations predict shorter survival in chondrosarcoma. J Cancer. 2018;9:998-1005. 29581779 10.7150/jca.22915 PMC5868167

[29] MacDonald IJ Lin CY Kuo SJ Su CM Tang CH. An update on current and future treatment options for chondrosarcoma. Expert Rev Anticancer Ther. 2019;19:773-786. 31462102 10.1080/14737140.2019.1659731

[30] Molenaar RJ Maciejewski JP Wilmink JW van Noorden CJF. Correction: wild-type and mutated IDH1/2 enzymes and therapy responses. Oncogene. 2018;37:5810. 30140044 10.1038/s41388-018-0455-1 PMC6887841

[31] Murugan AK Bojdani E Xing M. Identification and functional characterization of isocitrate dehydrogenase 1 (IDH1) mutations in thyroid cancer. Biochem Biophys Res Commun. 2010;393:555-559. 20171178 10.1016/j.bbrc.2010.02.095 PMC2838977

[32] Nakagawa M Sekimizu M Endo M Prognostic impact of IDH mutations in chondrosarcoma. J Orthop Sci. 2022;27:1315-1322. 34531086 10.1016/j.jos.2021.07.024

[33] Nazeri E Gouran Savadkoohi M Majidzadeh AK Esmaeili R. Chondrosarcoma: an overview of clinical behavior, molecular mechanisms mediated drug resistance and potential therapeutic targets. Crit Rev Oncol Hematol. 2018;131:102-109. 30293700 10.1016/j.critrevonc.2018.09.001

[34] Nota SP Braun Y Schwab JH van Dijk CN Bramer JA. The identification of prognostic factors and survival statistics of conventional central chondrosarcoma. Sarcoma. 2015;2015:623746. 10.1155/2015/623746 PMC4655064 26633939

[35] Pansuriya TC van Eijk R d'Adamo P Somatic mosaic IDH1 and IDH2 mutations are associated with enchondroma and spindle cell hemangioma in Ollier disease and Maffucci syndrome. Nat Genet. 2011;43:1256-1261. 22057234 10.1038/ng.1004 PMC3427908

[36] Polychronidou G Karavasilis V Pollack SM Huang PH Lee A Jones RL. Novel therapeutic approaches in chondrosarcoma. Future Oncol. 2017;13:637-648. 28133974 10.2217/fon-2016-0226

[37] Samuel AM Costa J Lindskog DM. Genetic alterations in chondrosarcomas - keys to targeted therapies? *Cell Oncol (Dordr*). 2014;37:95-105. 24458248 10.1007/s13402-014-0166-8 PMC13004474

[38] Shibata T Kokubu A Miyamoto M Sasajima Y Yamazaki N. Mutant IDH1 confers an in vivo growth in a melanoma cell line with BRAF mutation. Am J Pathol. 2011;178:1395-1402. 21356389 10.1016/j.ajpath.2010.12.011 PMC3069821

[39] Strotman PK Reif TJ Kliethermes SA Sandhu JK Nystrom LM. Dedifferentiated chondrosarcoma: a survival analysis of 159 cases from the SEER database (2001-2011). J Surg Oncol. 2017;116:252-257. 28420036 10.1002/jso.24650

[40] Suijker J Baelde HJ Roelofs H Cleton-Jansen AM Bovée JV. The oncometabolite D-2-hydroxyglutarate induced by mutant IDH1 or -2 blocks osteoblast differentiation in vitro and in vivo. Oncotarget. 2015;6:14832-14842. 26046462 10.18632/oncotarget.4024 PMC4558118

[41] van Praag Veroniek VM Rueten-Budde AJ Ho V Dijkstra PDS Fiocco M van de Sande MAJ. Incidence, outcomes and prognostic factors during 25 years of treatment of chondrosarcomas. Surg Oncol. 2018;27:402-408. 30217294 10.1016/j.suronc.2018.05.009

[42] Venneker S Szuhai K Hogendoorn PCW Bovée J. Mutation-driven epigenetic alterations as a defining hallmark of central cartilaginous tumours, giant cell tumour of bone and chondroblastoma. Virchows Arch. 2020;476:135-146. 31728625 10.1007/s00428-019-02699-2 PMC6968983

[43] Waitkus MS Diplas BH Yan H. Isocitrate dehydrogenase mutations in gliomas. Neuro Oncol. 2016;18:16-26. 26188014 10.1093/neuonc/nov136 PMC4677412

[44] WHO Classification of Tumours Editorial Board. Soft Tissue and Bone Tumours. International Agency of Research on Cancer; 2020.

[45] Xu W Yang H Liu Y Oncometabolite 2-hydroxyglutarate is a competitive inhibitor of α-ketoglutarate-dependent dioxygenases. Cancer Cell. 2011;19:17-30. 21251613 10.1016/j.ccr.2010.12.014 PMC3229304

[46] Xu X Zhao J Xu Z Structures of human cytosolic NADP-dependent isocitrate dehydrogenase reveal a novel self-regulatory mechanism of activity. J Biol Chem. 2004;279:33946-33957. 15173171 10.1074/jbc.M404298200

[47] Yan H Parsons DW Jin G IDH1 and IDH2 mutations in gliomas. N Engl J Med. 2009;360:765-773. 19228619 10.1056/NEJMoa0808710 PMC2820383

[48] Yang H Ye D Guan KL Xiong Y. IDH1 and IDH2 mutations in tumorigenesis: mechanistic insights and clinical perspectives. Clin Cancer Res. 2012;18:5562-5571. 23071358 10.1158/1078-0432.CCR-12-1773 PMC3897211

[49] Yen KE Schenkein DP. Cancer-associated isocitrate dehydrogenase mutations. Oncologist. 2012;17:5-8. 22234630 10.1634/theoncologist.2011-0429 PMC3267823

[50] Zhao S Lin Y Xu W Glioma-derived mutations in IDH1 dominantly inhibit IDH1 catalytic activity and induce HIF-1alpha. Science. 2009;324:261-265. 19359588 10.1126/science.1170944 PMC3251015

[51] Zhu GG Nafa K Agaram N Genomic profiling identifies association of IDH1/IDH2 mutation with longer relapse-free and metastasis-free survival in high-grade chondrosarcoma. Clin Cancer Res. 2020;26:419-427. 31615936 10.1158/1078-0432.CCR-18-4212 PMC6980683

